# The Role of miRNA-34a as a Prognostic Biomarker for Cirrhotic Patients with Portal Hypertension Receiving TIPS

**DOI:** 10.1371/journal.pone.0103779

**Published:** 2014-07-28

**Authors:** Christian Jansen, Hannah Eischeid, Jan Goertzen, Robert Schierwagen, Evrim Anadol, Christian P. Strassburg, Tilman Sauerbruch, Margarete Odenthal, Jonel Trebicka

**Affiliations:** 1 Department of Internal Medicine I, University of Bonn, Bonn, Germany; 2 Department of Pathology, University of Cologne, Cologne, Germany; University of Barcelona, Spain

## Abstract

**Background:**

Circulating miRNA-34a is increased in blood of patients with different liver diseases when compared to healthy controls. However, the origin of miRNA-34a and its possible relationship with hemodynamics and outcome in cirrhotic patients with portal hypertension is unknown. We analyzed the levels of miRNA-34a in cirrhotic patients with severe portal hypertension.

**Methods:**

We included 60 cirrhotic patients receiving TIPS for prevention of rebleeding and/or therapy-refractory ascites. miRNA-34a levels were measured using qPCR and normalized by SV-40 in the portal and hepatic venous blood of these patients taken at TIPS procedure. Hemodynamic and clinical parameters were assessed before TIPS and during follow-up.

**Results:**

Levels of miRNA-34a were higher in the hepatic vein than in the portal vein. Circulating miRNA-34a in the hepatic vein correlated with ALT, CHE and sodium excretion after TIPS. miRNA-34a showed no correlation with portal pressure, but its levels in the portal vein correlated inversely with the congestion index. Interestingly, the levels of miRNA-34a in the portal and hepatic vein showed inverse correlation with arterial pressure. Furthermore, levels of miRNA-34a in the hepatic vein had a predictive value for survival, but MELD, creatinine at short-time follow-up 14 days after TIPS-insertion and portal pressure after TIPS performed better.

**Conclusion:**

This study demonstrates for the first time, that miRNA-34a may originate to a large extent from the liver. Even though higher levels of miRNA-34a are possibly associated with better survival at long-term follow-up in cirrhotic patients with severe portal hypertension receiving TIPS, classical prognostic parameters predict the survival better.

## Introduction

Chronic liver injury of different etiologies leads to hepatic fibrosis, which might progress to end-stage liver disease. Portal hypertension often develops in these patients and is responsible for severe complications, such as variceal bleeding, refractory ascites and hepato-renal syndrome [1], [2]. Hyperdynamic circulation presenting with increased portosystemic shunting and arterial hypotension is a hallmark for the development of complications. Certain complications of portal hypertension can be treated using transjugular intrahepatic portosystemic shunt (TIPS), which leads to an immediate decompression of the portal venous system [2]. TIPS insertion might improve survival in well-selected patients with bleeding, refractory ascites and hepato-renal syndrome [3]–[6]. However, TIPS increases porto-systemic shunting and therefore leads to hepatic hypoperfusion, which can cause or aggravate hepatic encephalopathy. Therefore, careful selection of patients is mandatory, and new biomarkers might be useful in this regard.

Circulating micro ribonucleic acid (miRNA) have been increasingly evaluated as potential biomarkers in different disorders, including liver diseases [7]–[10] miRNA are small noncoding RNAs consisting of 19 to 25 nucleotides. miRNA modify the posttranscriptional processes of mRNA and regulate important cellular processes, such as differentiation, proliferation, metabolism and apoptosis [11]. It has been shown that circulating levels of miRNA-34a are associated with liver diseases. Since miRNA-34a has been associated also with other disorders besides liver diseases [12], [13], the major source of miRNA-34a remains unclear. Furthermore, there are hints that miRNA-34a might mirror the severity of liver disease. In human, as well as in mouse model of NASH, positive correlations of miR-34a levels with disease severity were already described [7]–[10]. Another study proposed a link between miR-34a and TGF-β in HBV positive patients, which is a major cytokine in development of liver fibrosis and progression of liver disease [14]. However, its role as a biomarker for severity of liver cirrhosis with portal hypertension remain unknown to date.

In the present study, circulating levels of miRNA-34a were measured in the portal and hepatic vein of cirrhotic patients with severe portal hypertension receiving TIPS. Our aim was to investigate the portentiol role of miRNA-34a as a predictor of complications and survival for cirrhotic patients receiving TIPS.

## Patients and Methods

### Patients and data collection

We retrospectively included 60 patients with liver cirrhosis underwent TIPS implantation. General clinical characteristics are shown in Table 1. Clinical, hemodynamic and biochemical parameters were assessed during the study. The patients signed a written inform consent for the procedures in the study. The local ethics committee of the University of Bonn approved the study (029/13).

**Table 1:** Clinical parameters of the patients (n = 60) at TIPS placement.

Parameters	Values	miRNA-34a>median	miRNA-34a<median
gender (female/male)	24/36	13/17	11/19
age median (range)	58 (40–77)	57. 5 (41–77)	57.5 (40–72)
etiology (alcohol/hepatitis/other)	45/7/8	21/4/5	24/3/3
Child category (A/B/C)	11/36/13	7/15/8	4/21/5
Child score median (range)	8 (5–12)	8 (5–11)	8 (5–12)
MELD score median (range)	10 (6–30)	9.5 (7–30)	10 (6–25)
indication (recurrent bleeding/refractory ascites/both)	26/27/7	14/13/3	12/14/4
esophageal varices (absent and Grade I/Grade II and III)	19/41	8/22	10/20
ascites (absent/Grade I/Grade II)	11/16/33	5/11/14	6/5/19
hepato-renal syndrome (absent/present)	46/14	25/5	21/9
hepatic encephalopathy (absent/present/undocumented)	21/10/29	12/7/11	9/3/18
antibiotic therapy (yes/no)	18/42	8/22	10/20
betablockers therapy (yes/no)	12/48	5/25	7/23

There was no significant difference in any variable between the groups.

MELD, model for end-stage liver disease.

### Study design

The patients received TIPS for therapy-refractory ascites (n = 27), recurrent bleeding (n = 26), or both indications (n = 7). TIPS (8–10 mm Wallstent, Boston Scientific, MA, USA) insertion was performed as previously described [3], [15], [16]. Portal and hepatic venous pressures were measured invasively using a pressure transducer system (Combitrans, Braun Melsung, Germany) and a multichannel monitor (Sirecust, Siemens, Germany). The difference between these pressures was defined as the portal hepatic venous pressure gradient (PHPG). Arterial pressure and heart rate were monitored non-invasively. Biochemical parameters, as well as portal and systemic hemodynamics, were measured and recorded at TIPS placement and during follow-up (Table 2 and 3). Biochemical parameters were analyzed using standard methods. In follow-up, portal vein flow and velocity, cross-sectional area of the portal vein were measured by ultrasound after a median of 14 days. We calculated the congestion index, which represents the ratio between the cross-sectional area and the blood flow velocity of the portal vein. The congestion index, first described by Moriyasu [17], mirrors best the congestion due to portal hypertension.

**Table 2:** Biochemical and hemodynamic parameters before TIPS insertion and at a short-term follow-up after a median of fourteen days (n = 60) compared using with Wilcoxon test.

	median	median	
Parameters	before TIPS	after TIPS	p-Value
bilirubin (mg/dL)	1.2	1.5	0.009
AST (U/L)	18	22	0.049
ALT (U/L)	17	20	0.009
γGT (U/L)	53	105	<0.001
INR	1.1	1.2	0.003
sodium (mmol/L)	135	137	0.002
serum creatinine (mg/dL)	1.00	0.93	0.048
BUN (mg/dL)	43	31	0.001
portal vein flow velocity (cm/s)	15	33	<0.001
portal vein flow (mL/min)	1,31	2,51	<0.001
PHPG (mmHg)	21	9	<0.001
Congestion index	0.101	0.046	<0.001

BUN, blood urea nitrogen; AST, aspartate aminotransferase; ALT, alanine aminotransferase; γGT, gamma-glutamyl transpeptidase; INR, International Normalized Ratio; PHPG, portal hepatic pressure gradient.

**Table 3:** Univariate time-to-event analysis of patient characteristics (including variables of table 1, 2 and 3). In the table are shown only significant variables.

		95% confidence interval		
Parameters	hazard ratio	low	upper	p-value
miRNA-34a in hepatic vein	0.889	0.775	1.019	0.091
MELD	1.129	1.053	1.21	0.001
creatinine in short-term follow-up	1.469	1.159	1.863	0.001
sodium before TIPS	0.857	0.794	0.925	<0.001
portal pressure before TIPS	1.056	1.005	1.109	0.03
portal pressure after TIPS	1.215	1.094	1.35	<0.001

MELD, model for end-stage liver disease.

### miRNA-34a isolation and quantification by real-time PCR

During the TIPS procedure, blood from the portal and hepatic veins to determine levels of miRNA-34a was collected from all patients as soon as the right branch of the portal vein was cannulated as previously described [3], [15], [16], [18]. Blood samples were centrifuged at 3000 rpm for 15 minutes at 4°C and stored at −80°C. RNA was isolated from serum samples using the Qiazol reagent following the instructions of the supplier (Qiagen, Hilden, Germany) as previously described. SV40-miRNA (Qiagen) was added to serum samples (2 pmol/200 µl) prior to the RNA isolation procedure for later normalization of circulating miRNA-34a levels, while RNA quantity was determined by A_260_-measurement using the ND-1000 NanoDrop spectrophotometer (NanoDrop, Wilmington, DE, USA) and quality was assessed by microcapillary electrophoresis (2100 BioAnalyser, Agilent Technologies, Waldbronn, Germany).

miRNA was analyzed by a two-step real-time PCR using the miScript-Reverse Transcription Kit and the miRNA-SYBR Green PCR Kit (Qiagen, Hilden, Germany). miRNA-34a and SV-40 primers used for cDNA synthesis and real-time PCR were selected and purchased from the GeneGlobe Search Center (Qiagen, Hilden, Germany). All steps were performed in triplicate and in agreement with the supplier's guidelines. For normalization of extracellular miRNA-34a levels, spike-in SV40-miRNA (Qiagen, Hilden, Germany) was used.

### Statistical analysis

In our study, we used the non-parametric Wilcoxon test to compare paired data and the Mann-Whitney test for unpaired comparisons. Correlations were analyzed with the Spearman correlation coefficient. Univariate time-to-event analysis was performed to identify parameters, which significantly predict survival. Cox-regression analysis (forward step-wise likelihood-quotient) using the significant predictors in the univariate analysis was performed to identify independent predictors of survival. Kaplan-Meier curves were used to analyze the survival rates of patients using the Log-rank test. Statistical analysis was performed by means of SPSS 22 for Windows SPSS Inc. Chicago,IL, USA).

## Results

### Clinical, biochemical and haemodynamic characteristics

The clinical characteristics of the included patients are listed in Table 1. The median age of the included patients (36 male, 24 female) was 58 years with a range of 40 to 77 years. The etiology of cirrhosis was alcohol in 45 patients, chronic hepatitis in seven patients, while eight patients had other diseases. Eleven patients presented with Child A, 36 with Child B and 13 with Child C liver cirrhosis. The MELD score showed a median of 10, with a range from 6–30 points. While patients had experienced hepato-renal syndrome, ten patients had experienced at least one episode of hepatic encephalopathy. The indication for TIPS was recurrent bleeding in 26 cases and refractory ascites in 27 cases. In seven patients, both indications for TIPS were present. Before TIPS, 47 patients were treated with non-selective beta-blocker, while 41 patients had received prior antibiotic therapy for infections (data not shown).

The short-term effects of TIPS on the biochemical parameters are shown in Table 2. TIPS led to significant increase of bilirubin (p = 0.009), AST (p = 0.049), ALT (p = 0.009), γGT (p<0.001) and INR (p = 0.003). Significant higher levels of sodium (p = 0.002) were found in patients after TIPS, when compared with levels before TIPS (Table 2), whereas levels of creatinine (p = 0.048) and BUN (p = 0.001) decreased after TIPS (Table 2).

Furthermore, after TIPS portal vein flow velocity (p>0.001) and portal vein flow (p<0.001) increased, while levels of portal vein pressure gradient (p<0.001), as well as the congestion index (p<0.001) decreased as marker of successfully TIPS intervention treatment of portal hypertension (Table 3).

Interestingly, the percentage of portal pressure gradient reduction after TIPS showed a median value of 56.7% (21–91.7). Patients with a greater reduction in portal pressure gradient than the median showed a better survival after 5 years (p = 0.035).

### Circulating levels of miRNA-34a derive from the liver and correlate with hepatic and renal parameters

**Figure 1:**
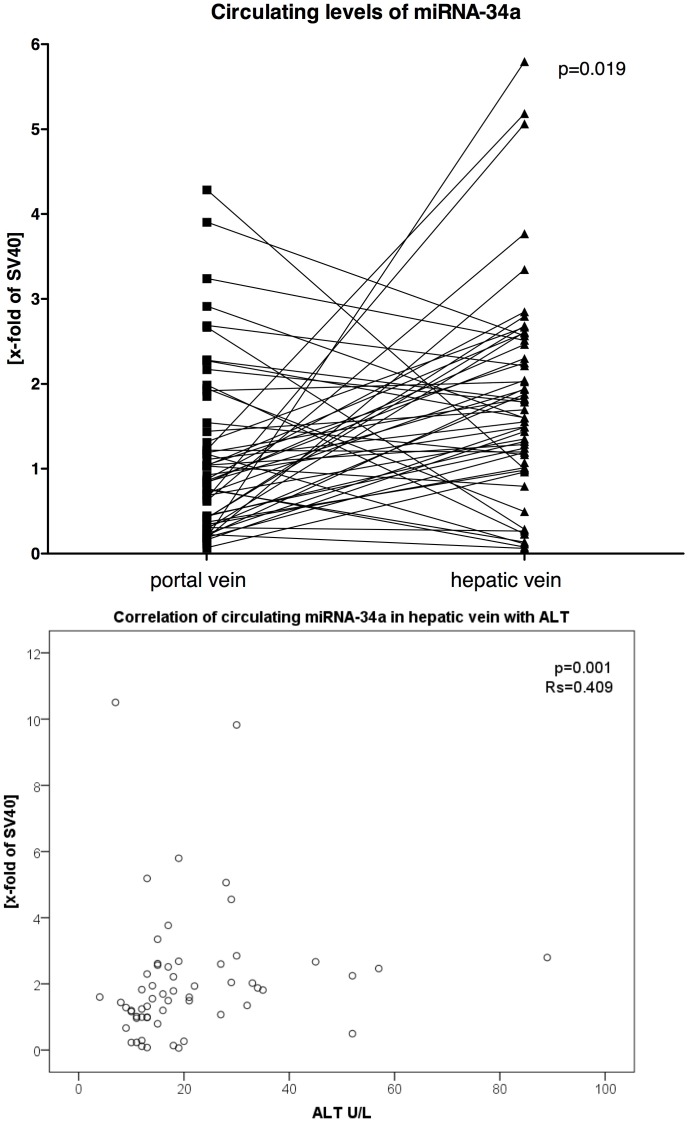
Serum levels of miRNA-34a in portal and hepatic vein before TIPS placement and its correlation with ALT-levels. (A) The levels of miRNA-34a measured in portal vein and hepatic vein before TIPS showed significant increase of miRNA-34a levels across the liver (p = 0.019). Data were shown paired and analyzed by Wilcoxon test. Of note, data of six patients lay outside of the shown range, and were not shown to increase readibility. (B) Levels of circulating miRNA-34a in the hepatic vein correlated significantly with ALT before TIPS (r_s_ = 0.409; p = 0.001). Data are presented using Spearman coefficient r_s_ and p-values. The levels of miRNA-34a were normalized to SV40 and are displayed as the x-fold of SV40.

Interestingly, the levels of miRNA-34a were significantly higher in the hepatic vein compared to the portal vein (p = 0.019) (Figure 1A).

We observed a relationship between miRNA-34a and hepatic and renal parameters. Levels of circulating miRNA-34a in the hepatic vein correlated significantly with ALT before TIPS (r_s_ = 0.409; p = 0.001; Figure 1B), CHE before TIPS (r_s_ = 0.372; p = 0.025) and sodium excretion in 24 h urine in the short-term follow-up after TIPS (r_s_ = 0.449; p = 0.005).

Furthermore, the levels of miRNA-34a in the portal and hepatic vein showed a significant inverse correlation with systolic (portal vein r_s_ = −0.316; p = 0.018 hepatic vein r_s_ = −0.271; p = 0.04) and diastolic (portal vein r_s_ = −0.275; p = 0.04; hepatic vein r_s_ = −0.262; p = 0.018) pressure measured non-invasively. There was no correlation of miRNA-34a with HPVG (data not shown). Additionally, the levels of miRNA-34a in the portal vein correlated inversely with the congestion index (r_s_ = −0.297; p = 0.0031) before TIPS. However, miRNA-34a measured in the portal vein showed inverse correlations with the cross sectional area in the portal vein before TIPS (r_s_ = −0.316; p = 0.02).

### The association of miRNA-34a measured in portal vein and hepatic vein with survival rates in patients receiving TIPS

**Figure 2:**
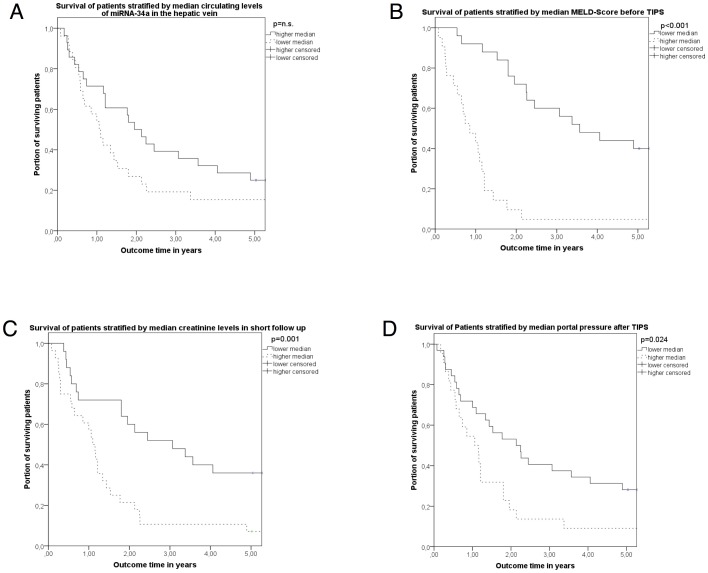
Survival 5 years after TIPS stratified using the median of circulating miRNA-34a (A) in the hepatic vein as well as MELD (B), creatinine in short-term follow-up (C) and portal pressure after TIPS (D). Patients were stratified for circulating miRNA-34a (A) measured in the hepatic vein, MELD-Score before TIPS (B), creatinine in short-term follow-up (C) and portal pressure after TIPS insertion (D) to higher and lower levels of the median of these parameters. Survival rates are shown using Kaplan-Meier plots and analyzed by log-rank test.

There was no significant correlation between survival and circulating levels of miRNA-34a in the portal or the hepatic vein. However, the patients with higher levels of miRNA-34a in the hepatic vein showed a tendency towards better survival in the long-term follow-up after TIPS (Table 4) (HR = 0.889; 95%CI: 0.775–1.019; p = 0.091; Table 4). Further parameters, such as MELD (HR = 1.129; 95%CI: 1.053–1.21; p = 0.001), creatinine in short-term follow-up (HR = 1.269; 95%CI: 1.159–1.863; p = 0.001), serum sodium before TIPS (HR = 0.857; 95%CI: 0.794–0.925; p<0.001), portal pressure before TIPS (HR = 1.056; 95%CI: 1.005–1.109; p = 0.03), and portal pressure after TIPS (HR = 1.215; 95%CI: 1.094–1.35; p<0.001) were identified as predictors of survival using the univariate time-to-event analysis (Table 4, Figure 2). When stratifying this collective of patients using the median value of circulating miRNA-34a levels in the hepatic vein, patients with higher levels of miRNA-34a tended towards better survival (Figure 2 A).

**Table 4:** Cox regression analysis (forward step-wise likelihood-quotient) using the significant variable from univariate analysis (table 4) to predict survival.

		95% confidence interval		
Parameters	Hazard ratio	lower	upper	p-value
MELD	5.0	1.7	14.9	0.003
creatinine in short-term follow-up	103	2.6	3,970	0.013
portal pressure after TIPS	1.35	1.04	1.75	0.023

MELD, model for end-stage liver disease.

Multivariable Cox regression analysis of the significant variables identified MELD (HR = 5.1; 95%CI: 1.7–15; p = 0.003), creatinine in short-term follow-up (HR = 103; 95%CI: 2.6–3,971; p = 0.013) and portal pressure after TIPS (HR = 1.4; 95%CI: 1.04–1.75; p = 0.023) as independent predictor of survival (Table 4).

## Discussion

This study demonstrates for the first time that miRNA-34a originates to a large extent from the liver and that levels of circulating miRNA-34a in hepatic vein correlated with parameters of hepatic dysfunction. Interestingly, miRNA-34a levels in the portal vein correlated inversely with portal venous congestion. Also, higher levels of circulating miRNA-34a in the hepatic vein might have a predictive value for survival in patients receiving TIPS.

Liver cirrhosis with portal hypertension is a major cause of morbidity and mortality world-wide [19]–[31]. TIPS is a widely accepted and used therapy for portal hypertension. TIPS placement decompresses the portal venous system. It reduces portal venous perfusion, with higher risk of hepatic encephalopathy and liver failure [28], [30]–[33]. Therefore, careful selection of patients receiving TIPS is required and new markers for the predictive of complications after TIPS are needed.

Circulating miRNAs have been discussed as biomarkers for different disorders. This study demonstrates for the first time that miRNA-34a originates to a large extent from the liver. In the setting of TIPS-insertion, blood from the portal vein and from the hepatic vein was withdrawn simultaneously and the gradient showed a significant increase of miRNA-34a across the liver. This finding is supported by the positive correlation of the levels in the hepatic vein with aminotransferases and cholinesterase.

Interestingly, miRNA-34a in the portal venous compartment showed an inverse correlation with the cross-sectional area of portal vein measured by ultrasound and the portal congestion index. The congestion index, first described by Moriyasu, is calculated from parameters measured by ultrasound. This calculated index mirrors best the congestion due to portal hypertension [17]. The inverse correlation of miRNA-34a level in the portal vein with the portal venous congestion may be explained by lower hepatic perfusion with increasing congestion of the portal vein. Even though the systemic circulation was not assessed invasively at TIPS-procedure, one might be speculated that the decreased hepatic perfusion compared to systemic circulation could be the reason for the decreased release of miRNA-34a into the circulation and therefore lower levels in the arterial blood and consequently in the portal venous blood. The relationship of miRNA-34a and organ perfusion is further supported by the finding that systemic arterial pressures correlated inversely with levels of miRNA-34a in both vascular compartments. Hence, an increased splanchnic perfusion pressure in the situation of portal hypertension means higher portal venous congestion with consequently lower miRNA-34a levels, as found for the congestion index.

Although, levels of miRNA-34a in the portal vein possibly explain the portal vein congestion in the situation of severe portal hypertension, these levels showed no predictive value after portal venous decompression using TIPS. In contrast, the levels of circulating miRNA-34a measured in the hepatic vein tended to predict survival in these patients in univariate analysis. This finding might be explained by the correlation of these levels in the hepatic vein with ALT and CHE, which possibly mirror conserved liver function. Indeed, when stratifying the patients using the median of the miRNA-34a levels in the hepatic vein, patients with higher circulating levels of miRNA-34a had a better survival. After TIPS, patients with better conserved liver function showed a better survival, since MELD was an independent predictor of mortality in multivariate analysis in our collective besides renal function and portal pressure after TIPS. Interestingly, circulating miRNA-34a correlated with sodium excretion after TIPS as a marker of restored renal function. Thus, both the preserved hepatic function before TIPS and the restored renal function after TIPS may play a role in survival and are associated with the hepatic venous levels of miRNA-34a. Therefore, levels of miRNA-34a in the hepatic vein might represent a valuable biomarker for the outcome of patients receiving TIPS, but not in the same degree as MELD; creatinine in short-term follow-up and portal pressure after TIPS. In this collective of patients MELD, creatinine and portal pressure are more appropriate to predict outcome than the levels of miRNA-34a.

Although our patient collective was very well characterized before and after TIPS with a long-term follow up, our study has several limitations. First, the extraction of miRNA was performed phenol-based and therefore, we did not distinguish between vesicle bound and free miRNA. The analysis of the patient data was retrospective and could not elucidate the cause of death in these patients. Despite these limitations, the studied cohort was consistent with other studies, since MELD, creatinine in follow up and portal pressure after TIPS were independent predictors of mortality.

As already shown by Malinchoc et al, MELD-Score is the best predictor of survival in patients receiving TIPS [34]. However, we could not confirm the role of bilirubin or age as prognostic marker in these patients as described previously [4], [35]. However, the level of portal pressure gradient seems to be a good marker of survival as it has been described for HVPG [36].

In conclusion, this study demonstrates for the first time that miRNA-34a might originate to a large extent from the diseased liver and hepatic venous level of miRNA-34a might be a predictor of outcome in cirrhotic patients receiving TIPS. Future studies are needed to confirm these findings.
